# Reproducibility of Left Ventricular Dyssynchrony Indices by Three-Dimensional Speckle-Tracking Echocardiography: The Impact of Sub-optimal Image Quality

**DOI:** 10.3389/fcvm.2019.00149

**Published:** 2019-10-10

**Authors:** Lamia Al Saikhan, Chloe Park, Alun D. Hughes

**Affiliations:** ^1^Department of Cardiac Technology, College of Applied Medial Sciences, Imam Abdulrahman Bin Faisal University, Dammam, Saudi Arabia; ^2^MRC Unit for Lifelong Health and Ageing at UCL, Department of Population Science and Experimental Medicine, UCL Institute of Cardiovascular Science, London, United Kingdom

**Keywords:** 3D speckle tracking, left ventricular dyssynchrony, image quality, test-retest reliability, reproducibility

## Abstract

**Background:** 3D speckle-tracking echocardiography (3D-STE) is a novel method to quantify left ventricular (LV) mechanical dyssynchrony. 3D-STE is influenced by image quality, but studies on the magnitude of its effect on 3D-STE derived LV systolic dyssynchrony indices (SDIs) and their test-retest reproducibility are limited.

**Methods:** 3D-STE was performed in two groups, each comprising 18 healthy volunteers with good echocardiographic windows. In study 1, optimal and inferior-quality images, by intentionally poor echocardiographic technique, were acquired. In study 2, sub-optimal quality images were acquired by impairing ultrasound propagation using neoprene rubber sheets (thickness 2, 3, and 4 mm) mimicking mildly, moderately, and severely impaired images, respectively. Measures (normalized to cardiac cycle duration) were volume- and strain-based SDIs defined as the standard deviation of time to minimum segmental values, and volume- and strain-derived dispersion indices. For both studies test-retest reproducibility was assessed.

**Results:** Test-retest reproducibility was better for most indices when restricting the analysis to good quality images; nevertheless, only volume-, circumferential strain-, and principal tangential strain-derived LV dyssynchrony indices achieved fair to good reliability. There was no evidence of systematic bias due to sub-optimal quality image. Volume-, circumferential strain-, and principal tangential strain-derived SDIs correlated closely. Radial strain- and longitudinal strain-SDI correlated moderately or weakly with volume-SDI, respectively.

**Conclusions:** Sub-optimal image quality compromised the reliability of 3D-STE derived dyssynchrony indices but did not introduce systematic bias in healthy individuals. Even with optimal quality images, only 3D-STE indices based on volume, circumferential strain and principal tangential strain showed acceptable test-retest reliability.

## Introduction

Synchronous contraction is important for overall left ventricular (LV) systolic performance (1). Widening of the QRS duration on ECG is widely used as a marker of intra-ventricular dyssynchrony. However, LV mechanical dyssynchrony (LVMD) (i.e., dyssynchronous contraction and relaxation of the myocardium) may occur in the absence of ECG evidence (2). LVMD assessed using different imaging modalities has been shown to be an independent predictor of poor prognosis in cardiac disease (3–9). Hence, it may have advantages in guiding cardiac resynchronization therapy (CRT), although current guidelines are still based on electrical dyssynchrony criteria (10).

LVMD can be measured by several echocardiographic imaging modalities such tissue-Doppler imaging (TDI), 2D speckle-tracking echocardiography (STE), 3D echocardiography (3DE), and 3D-STE (11). TDI-based dyssynchrony indices do not improve patient selection for CRT (12), but 2D-STE based dyssynchrony indices have demonstrated added value in selecting potential candidates for CRT even when QRS duration is borderline (13, 14). Despite promising results, 2D-STE is limited by the requirement for non-simultaneous measurements introducing beat-to-beat variability (15). Furthermore, measurements are restricted to a single plane, and complex LV dyssynchrony patterns may be overlooked by a 2D method (13, 15).

3D-STE has also emerged as a promising method for quantifying LVMD, with 3DE systolic dyssynchrony index (SDI-volume) having been proposed as a useful measure to assess LVMD and guide CRT (16). In addition to SDI-volume, parameters calculated from different myocardial strain vectors [i.e., longitudinal strain (LS), radial strain (RS), circumferential strain (CS), and recently area strain (AS)] have been suggested as potentially useful measures of myocardial mechanics in LVMD (15).

For a test to be useful it must be reproducible, i.e., show acceptable variability between measures (good reliability) and demonstrate no systematic differences between measurement occasions (no bias) (17). Good reproducibility of 3DE dyssynchrony indices has been reported in a meta-analysis of several studies (16), but one study reported that poor image quality impaired reliability of 3D dyssynchrony by ~12–21% (18). However, these studies (15, 16, 19–22) including the study of image quality (18) were based on re-reading the same scans. Re-reading scans may substantially underestimate scan-rescan reliability and has limited ability to detect bias; test-retest reproducibility is usually more relevant to chronic studies (17). The effect of image quality on test-retest reproducibility of 3D-STE derived dyssynchrony indices has not been reported and evidence on systematic bias related to image quality is limited. It is difficult to assess this bias in observational studies examining correlations between reliability and scan quality since these associations are confounded by factors such as age, adiposity, or other (potentially unmeasured) risk factors that may jointly influence scan quality and dyssynchrony measures (23). Experimental modification of image quality avoids this confounding but evidence on the controlled effect of changing image quality on 3D-STE derived dyssynchrony indices is lacking.

We aimed therefore to (1) quantitate the impact of intentionally distorted image quality on reliability and bias of LV dyssynchrony indices by 3D-STE; (2) assess the association between SDI-volume and strain-based LV SDIs. By design, the study was conducted in healthy individuals with good echocardiographic windows since this allowed us to achieve a realistic degree of intentional image impairment to compare with good quality reference images. Some of these data have been presented previously in abstract form (24, 25).

## Methods

### Study Population

Two prospective sub-studies (study 1 and study 2; conducted at different times) were performed. In study 1, 23 healthy individuals with no previous cardiac medical history were recruited to undergo 3DE. Only individuals with excellent/optimal echocardiographic windows were included in these studies, so 5 individuals were excluded in study 1 due to sub-optimal echocardiographic windows, leaving a final sample size of 18. In study 2 study, an additional 21 healthy individuals were recruited to further quantify the impact of sub-optimal image quality. Eighteen participants were finally included after excluding 3 participants with sub-optimal echocardiographic windows. The institutional review board approved the study protocol and informed consent was obtained from all participants at the time of examination.

### Image Acquisition and Analysis

3DE examination was perfumed using an EPIQ7 ultrasound machine (Philips Medical Systems, Andover, MA) equipped with a X5-1 Xmatrix-array transducer by an experienced, British Society of Echocardiography accredited sonographer as previously described (26). Following a standard protocol, LV-focused 4 wedged-shaped sub-volumes were acquired over 4 consecutive cardiac cycles during a single breath-hold from the apical window using harmonic imaging and multiple-beat 3DE mode (27). During the acquisition, special care was taken to include the entire LV cavity within the pyramidal sector volume.

**Figure 1:**
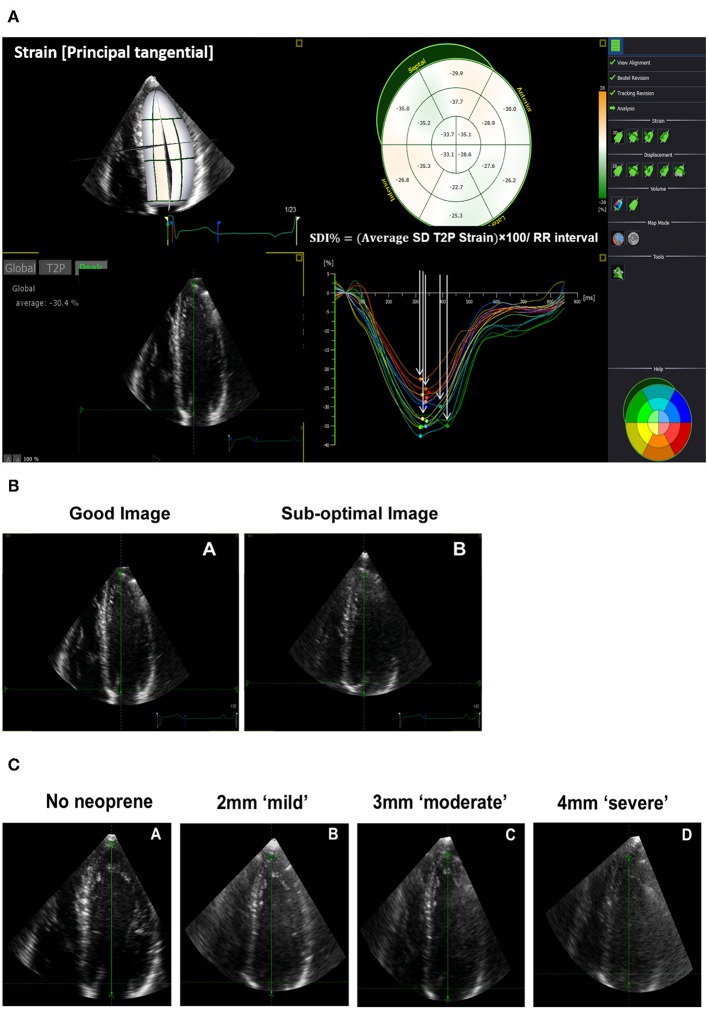
Three-dimensional echocardiographic (3DE) strain derived systolic dyssynchrony index (SDI) and impaired quality 3DE images. **(A)** SDI derived from principle tangential strain. **(B)** An example of a good and sub-optimal 3DE image quality obtained from the same participant in study 1. **(C)** An example of a 3DE with an optimal quality reference (no neoprene), mild (2 mm neoprene), moderate (3 mm neoprene), and severe (4 mm neoprene) impairment of 3DE image quality obtained from the same participant in study 2.

In study 1, two gated wide-angled 3DE LV full-volume datasets were obtained per participant. The acquisition of the first dataset was performed according to EAE/ASE recommendations (27). Machine settings including gain, sector width, and depth were adjusted by the operator to maximize the quality of images ensuring clear visualization of LV endocardial borders and avoiding echo drop-out. A good 3DE image was defined as clear visualization of the endocardium in all 16-segments in both end-diastolic and end-systolic frames. The quality of the second dataset was impaired by using an intentionally sub-optimal echocardiographic technique. This was achieved by a combination of scanning the participants while laying supine resulting in more distance between the transducer and the heart, and absence of gel to create an air-tissue interface initiating multiple reflections and acoustic shadowing artifacts. This resulted in echo drop out, shadow artifacts, or poor visualization of the endocardium A sub-optimal 3DE image was defined as the presence of at least one of the following (Figure 1B):

1. Poor visualization of the endocardium of up to 7 segments throughout the cardiac cycle in a 16-segment LV model.
2. The presence of echo dropout.
3. Shadow artifacts.

An identical acquisition protocol (optimal and sub-optimal images) under the same conditions was repeated on the same day (typically within 1 h of the original scan) to assess the test-retest reproducibility of optimal and sub-optimal images.

In study 2, the quality of the 3DE images was impaired by attenuating ultrasound propagation in a graded and reproducible manner to better quantitate the impact of sub-optimal image quality. In achieving this, we used three sheets of ultrasound-attenuating material called neoprene rubber with different thicknesses (2, 3, and 4 mm) to mimic mild, moderate, and severe impairment in image quality, respectively (Figure 1C). Neoprene is a polymer of chloroprene, 2 chloro-1, and 3-butadiene. We chose it as many of its acoustic properties are similar to soft biological tissues, it is durable, and it has a comparatively high attenuation coefficient (28, 29). Each sheet of neoprene rubber was placed between the skin and the transducer with ultrasound gel on both sides after which images were captured. In total, 4 gated 3DE LV full-volume datasets including an optimal quality reference image (no neoprene) were acquired per participant (Figure 1C). We avoided images with stitching artifacts and ensured good quality ECG signals. We maintained a constant frame rate between scans with a minimum acquisition rate of 18 frames/second (30) and optimal machine settings.

Image analysis was perfumed using 4D LV-Analysis software (TomTec Imaging Systems GmbH, Germany, 2015) by a single blinded reader. In total, 4 datasets per participant for each sub-study were analyzed. To quantify read-to-read variability a second read of scan was performed ~2–3 months after the initial read. At the start of each analysis, three standard apical views and one short-axis view were automatically selected and displayed by the software. The longitudinal axis of the LV in all apical views were further aligned manually if needed using the mitral valve annulus and the apex as anatomical landmarks. The LV endocardial borders in all three apical views were then automatically defined by the software at end-diastole and tracked throughout the cardiac cycle in 3D space from which the 3D LV endocardial shell was constructed. Further, manual adjustments were kept as minimal as possible. The software then subdivided the LV into 16 segments using a standard model (31) and provided curves as well as maps of global and segmental volumetric and deformation indices.

LV dyssynchrony indices were the following (Figure 1A):

- Volume-based SDI, defined as the SD of time to minimum segmental volumes over 16-LV segments.
- Strain-based SDI, calculated as the SD of time to peak segmental strain over 16 LV-segments from LS, CS, RS, and principle tangential strain (PTS) which is a composite 3D measure of CS and LS.
- Volume- and strain-derived dispersion indices (Di), calculated as the difference between minimum and maximum time to peak of segmental volume/strain values over 16-LV segments.

All indices were normalized to cardiac cycle length and reported as %.

### Statistical Analysis

Continuous variables are presented as mean ± SD. Categorical variables are presented as counts and percentages. Analysis was performed using mixed linear modeling method to assess bias and reliability (fixed effects: scan occasion and image quality, random effect: participant identity). Test-retest reliability was quantified by intraclass correlation coefficients (ICC). Reliability was classified as follows: ICC < 0.4 = poor, 0.4 ≥ ICC < 0.75 = fair to good, and ICC ≥ 0.75 = excellent (32). Measurement error was assessed as the standard error of estimates derived from the mixed linear modeling as advocated by Popović and Thomas (33). Bias was assessed using Bland Altman analysis and presented as the mean difference with limits of agreement between scans of different image quality. Intra-observer reproducibility based on re-reading the same (good quality) scans and was performed by the first reader (LA) blinded to the original measurements after 2–3 months interval. Over the same interval, inter-observer reproducibility was also performed by a second reader (CP) blinded to first reader's measurements. Linear correlations were analyzed and summarized using Pearson's correlation coefficient, *r*. The sample size was chosen to ensure a lower limit of the one-sided confidence interval ≤ 0.15 assuming a ICC = 0.8 (34). With this sample size we could also detect a bias ≥ 1SD (alpha = 0.05) with 96% power. All analyses were performed in Stata version 15.1 (StataCorp LLC, USA).

## Results

### Baseline Characteristics

Characteristics of participants in study 1 and 2 are summarized in Table 1. Individuals in study 1 were 28 ± 6 years old and 10 (55.5%) were men. Individuals in study 2 were 31 ± 6 years old and 15 (83.3%) were men. In study 1, the frame rate (per cycle) was 21 ± 4 (SD) and 21 ± 3 (SD) for good and sub-optimal quality images, respectively. For study 2, frame rate (per cycle) was 21 ± 3 (SD), 21 ± 3 (SD), 21 ± 3 (SD), and 21 ± 3 (SD) for the optimal, mildly, moderately, and severely impaired quality images, respectively.

**Table 1:** Study population characteristics.

	**Study 1 (*n* = 18)**	**Study 2 (*n* = 18)**
Age, years	28 ± 6	31 ± 6
Male, number (%)	10 (55.5%)	15 (83.3%)
Systolic blood pressure, mmHg	118.2 ± 8.6	123.2 ± 9.2
Diastolic blood pressure, mmHg	73.5 ± 7.3	77.3 ± 9.5
Heart rate, bpm	72 ± 13.8	69.1 ± 14.2
Height, cm	169.9 ± 9.4	172.2 ± 8.7
Weight, kg	70.9 ± 16.1	73.0 ± 8.1
Body mass index, kg/m^2^	24.5 ± 5.8	24.7 ± 3.6
Body surface area, m^2^	1.8 ± 0.21	1.8 ± 0.12
**3D-STE derived LV measures**		
3D EF, %	57.3 ± 3.3	55.4 ± 2.5
3D EDV, ml/m^2^	67.8 ± 8.9	74.9 ± 13.2
3D ESV, ml/m^2^	28.9 ± 4.4	33.5 ± 6.7
3D SV, ml	70.4 ± 9.9	77.1 ± 13.1
3D LV mass, g	127.7 ± 17.7	129.8 ± 14.1
SDI _volume_, %	5.4 ± 1.1	4.4 ± 0.9
CS-SDI, %	5.4 ± 1.5	4.8 ± 1.4
LS-SDI, %	2.1 ± 0.6	1.7 ± 0.7
RS-SDI, %	3.7 ± 1.1	3.3 ± 0.9
PTS-SDI, %	4.3 ± 1.1	3.3 ± 1.0
Di _volumes_, %	16.2 ± 2.9	14.3 ± 2.6
CS-Di, %	17.6 ± 4.6	16.7 ± 5.7
LS-Di, %	7.4 ± 2.1	5.9 ± 2.3
RS-Di, %	12.6 ± 2.9	11.2 ± 2.7
PTS-Di, %	14.8 ± 3.9	11.7 ± 3.1

*Data are mean ± standard deviation or number (%). CS, circumferential strain; Di, dispersion (difference between minimum and maximum time to peak of measure over 16-LV segments normalized to cardiac cycle duration); EDV, end-diastolic volume; EF, ejection fraction; ESV, end-systolic volume; LS, longitudinal strain; LV, left ventricle; PTS, principle tangential strain; RS, radial strain; SDI, systolic dyssynchrony index; SV, stroke volume; 3D-STE, three-dimensional speckle tracking echocardiography*.

### Test-Retest and the Impact of Image Quality on Reliability and Bias

Under optimal conditions (i.e., good quality images), only volume, CS and PTS 3D-STE derived dyssynchrony indices achieved fair to good test-retest reliability, whereas LS and RS derived dyssynchrony indices showed poor test-retest reliability (Table 2). Reduced 3DE image quality impaired the reliability of 3D-STE derived LV dyssynchrony indices with test-retest reliability being poor for all indices when images were sub-optimal (Table 2).

**Table 2:** Test-retest reliability.

	**ICC**	
	**Optimal images**	**Sub-optimal images**
SDI _volume_, %	0.69	0.20
CS-SDI, %	0.73	0.36
LS-SDI, %	0.40	0.42
PTS-SDI, %	0.52	0.16
RS-SDI, %	0.28	0.07
Di _volumes_, %	0.71	0.04
CS-Di, %	0.69	0.23
LS-Di, %	0.29	0.46
PTS-Di, %	0.60	0.24
RS-Di, %	0.16	0.04

*Abbreviations are as in Table 1 in addition to ICC, intraclass correlation coefficient*.

There was no evidence of systematic bias due to sub-optimal image quality in any of 3D-STE derived LV dyssynchrony indices in study 1 (Table 3). In study 2 using neoprene sheets, there was evidence of a small degree of systematic underestimation in volume derived dyssynchrony indices with increasingly poor image quality (Table 4). Bland and Altman analysis by image quality is shown in Table S1 and Figure S1. Suboptimal image analyses showed higher mean difference (±SD) and wider limit of agreement for all 3D-STE drive LV dyssynchrony indices compared to good image analyses.

**Table 3:** Comparison of 3D-STE derived LV dyssynchrony indices by image quality (Study 1).

	**Mean (95% CI)**		**Bias**	
	**Optimal**	**Suboptimal**	**Mean Δ ± SEM (95% CI) [optimal – suboptimal]**	***P*_**Bon**_**
SDI _volume_, %	5.4 (4.9, 5.8)	4.9 (4.5, 5.4)	0.41 ± 0.21 (0.002, 0.82)	0.25
CS-SDI, %	5.4 (4.8, 5.9)	5.4 (4.8, 5.9)	−0.02 ± 0.23 (−0.47, 0.43)	>0.9
LS-SDI, %	2.0 (1.7, 2.4)	2.2 (1.8, 2.6)	−0.16 ± 0.23 (−0.62, 0.30)	>0.9
PTS-SDI, %	4.3 (3.9, 4.8)	3.8 (3.3, 4.2)	0.58 ± 0.24 (0.12, 1.05)	0.07
RS-SDI, %	3.9 (3.5, 4.3)	3.7 (3.3, 4.1)	0.19 ± 0.22 (−0.25, 0.62)	>0.9
Di _volumes_, %	16.3 (15.0, 17.6)	15.7 (14.4, 16.9)	0.67 ± 0.63 (−0.58, 1.9)	>0.9
CS-Di, %	17.6 (15.5, 19.6)	18.5 (16.4, 20.6)	−0.96 ± 0.84 (−2.6, 0.69)	>0.9
LS-Di, %	7.2 (6.0, 8.5)	7.8 (6.6, 9.1)	−0.60 ± 0.78 (−2.1, 0.93)	>0.9
PTS-Di, %	14.6 (13.1, 16.1)	13.3 (11.8, 14.7)	1.3 ± 0.69 (−0.01, 2.7)	0.26
RS-Di, %	13.0 (11.7, 14.3)	12.8 (11.5, 14.1)	0.19 ± 0.76 (−1.3, 1.7)	>0.9

*Data are means (95% confidence intervals), P_Bon_ are Bonferroni adjusted P values. Abbreviations as in Table 1*.

**Table 4:** Comparison of 3D-STE derived LV dyssynchrony indices by image quality (Study 2).

	**Extent of bias relative to the reference Mean Δ ± SEM (95% CI)**			***P* (trend)**	**Mean (95% CI)**			
	**Mild**	**Moderate**	**Severe**		**Reference**	**Mild**	**Moderate**	**Severe**
SDI _volume_, %	−0.02 ± 0.32 (−0.6, 0.6)	−0.79 ± 0.32 (−1.4, −0.17)	−1.1 ± 0.32 (−1.7, −0.5)	<0.0001	4.4 (3.9, 4.9)	4.4 (3.9, 4.9)	3.6 (3.1, 4.2)	3.3 (2.8, 3.8)
CS-SDI, %	0.06 ± 0.51 (−0.94, 1.0)	−0.25 ± 0.51 (−1.2, 0.74)	0.59 ± 0.51 (−0.41, 1.6)	0.37	4.8 (3.9, 5.6)	4.8 (4.0, 5.7)	4.5 (3.7, 5.4)	5.4 (4.5, 6.2)
LS-SDI, %	−0.18 ± 0.7 (−1.5, 1.2)	−0.32 ± 0.7 (−1.7, 1.0)	0.90 ± 0.7 (−0.46, 2.3)	0.25	1.7 (0.69, 2.6)	1.5 (0.51, 2.5)	1.3 (0.37, 2.3)	2.6 (1.6, 3.5)
PTS-SDI, %	0.08 ± 0.76 (−1.4, 1.6)	−0.49 ± 0.76 (−1.9, 1.0)	0.48 ± 0.76 (−1.0, 1.9)	0.71	3.3 (2.2, 4.4)	3.4 (2.3, 4.5)	2.8 (1.7, 3.9)	3.8 (2.7, 4.9)
RS-SDI, %	−0.08 ± 0.59 (−1.2, 1.1)	−0.7 ± 0.59 (−1.8, 0.45)	0.38 ± 0.59 (−0.78, 1.5)	0.78	3.3 (2.4, 4.2)	3.2 (2.3, 4.1)	2.6 (1.7, 3.5)	3.7 (2.8, 4.5)
Di _volumes_, %	−0.63 ± 0.59 (−2.5, 1.2)	−1.9 ± 0.59 (−3.8, 0.01)	−2.2 ± 0.59 (−4.1, −0.3)	0.011	14.3 (12.7, 15.9)	13.7 (12.0, 15.3)	12.4 (10.8, 14.1)	12.1 (10.5, 13.8)
CS-Di, %	−0.74 ± 2.0 (−4.7, 3.2)	0.09 ± 2.0 (−3.9, 4.1)	3.6 ± 2.0 (−0.31, 7.6)	0.07	16.7 (13.5, 19.9)	15.9 (12.7, 19.1)	16.8 (13.6, 19.9)	20.3 (17.1, 23.5)
LS-Di, %	−0.63 ± 2.1 (−4.8, 3.5)	−1.2 ± 2.1 (−5.3, 3.0)	2.7 ± 2.1 (−1.5, 6.9)	0.27	5.9 (2.9, 8.8)	5.2 (2.2, 8.2)	4.7 (1.7, 7.7)	8.6 (5.6, 11.6)
PTS-Di, %	−0.03 ± 2.1 (−4.2, 4.1)	−1.5 ± 2.1 (−5.7, 2.6)	1.5 ± 2.1 (−2.7, 5.7)	0.66	11.7 (8.6, 14.8)	11.7 (8.5, 14.8)	10.2 (7.0, 13.3)	13.2 (10.1, 16.4)
RS-Di, %	−0.67 ± 1.9 (−4.3, 3.0)	−2.1 ± 1.9 (−5.8, 1.6)	1.9 ± 1.9 (−1.7, 5.6)	0.47	11.2 (8.4, 13.9)	10.5 (7.7, 13.3)	9.1 (6.3, 11.9)	13.1 (10.3, 15.9)

*Data are means (95% confidence intervals). Abbreviations as in Table 1*.

Intra-observer reproducibility based on re-reading the same (good quality) scans showed good to excellent reproducibility of all dyssynchrony indices except for LS derived dyssynchrony indices (Table 5). Reproducibility was fair to good for LS-SDI, but poor for LS-Di. Inter-observer reproducibility was excellent for volume-based dyssynchrony indices, and CS and RS derived SDIs, but only fair to good for PTS and LS derived SDIs (Table 6). Overall, strain derived SDI indices showed better inter-observer reproducibility than strain-derived dispersion indices (Table 6). Only CS-Di and RS-Di showed fair to good inter-observer reproducibility, being poor for LS-Di and PTS-Di (Table 6).

**Table 5:** Intra-observer reproducibility based on re-reading the good quality scans (*N* = 10).

	**Mean (95% CI)**		**Bias**	**ICC**
	**1st reading**	**2nd reading**	**Mean Δ ± SEM (95% CI)**	
SDI _volume_, %	5.1 (4.3, 5.8)	5.03 (4.3, 5.8)	−0.03 ± 0.06 (−0.16, 0.08)	0.98
CS-SDI, %	5.0 (4.0, 5.9)	5.3 (4.4, 6.2)	0.31 ± 0.19 (−0.08, 0.69)	0.87
LS-SDI, %	2.0 (1.6, 2.3)	1.8 (1.5, 2.1)	−0.17 ± 0.11 (−0.39, 0.04),	0.69
PTS-SDI, %	4.0 (3.4, 4.7)	4.3 (3.5, 5.0)	0.24 ± 0.08 (0.08, 0.4)	0.96
RS-SDI, %	3.8 (3.0, 4.6)	3.8 (3.0, 4.6)	−0.012 ± 0.08 (−0.17, 0.14)	0.97
Di _volumes_, %	15.3 (13.8, 16.8)	15.4 (13.6, 17.1)	0.04 ± 0.33 (−0.62, 0.69)	0.88
CS-Di, %	16.6 (13.4, 19.8)	16.7 (14.1, 19.3)	0.07 ± 0.9 (−1.7, 1.8)	0.73
LS-Di, %	7.1 (5.9, 8.2)	6.3 (5.5, 7.2)	−0.73 ± 0.52 (−1.7, 0.28)	0.26
PTS-Di, %	13.6 (11.8, 15.3)	14.1 (12.2, 16.1)	0.57 ± 0.2 (0.02, 1.1)	0.94
RS-Di, %	12.9 (10.9, 14.9)	12.5 (10.7, 14.4)	−0.38 ± 0.23 (−0.84, 0.08)	0.96

*Data are means (95% confidence intervals). Abbreviations as in Table 1*.

**Table 6:** Inter-observer reproducibility based on re-reading the good quality scans (*N* = 10).

	**Mean (95% CI)**		**Bias**	**ICC**
	**1st observer**	**2nd observer**	**Mean Δ ± SEM (95% CI)**	
SDI _volume_, %	5.1 (4.3, 5.8)	5.1 (4.3, 5.9)	0.05 ± 0.08 (−0.11, 0.21)	0.96
CS-SDI, %	5.0 (4.0, 5.9)	6.1 (4.5, 7.7)	1.1 ± 0.37 (0.40, 1.9)	0.77
LS-SDI, %	1.9 (1.7, 2.3)	1.9 (1.5, 2.3)	−0.10 ± 0.16 (−0.41, 0.21)	0.50
PTS-SDI, %	4.0 (3.4, 4.7)	4.4 (3.2, 5.6)	0.34 ± 0.35 (−0.33, 1.0)	0.64
RS-SDI, %	3.8 (3.0, 4.6)	3.7 (2.8, 4.6)	−0.08 ± 0.23 (−0.53, 0.37)	0.79
Di _volumes_, %	15.3 (13.8, 16.8)	15.4 (13.6, 17.1)	0.05 ± 0.36 (−0.66, 0.76)	0.86
CS-Di, %	16.6 (13.4, 19.8)	21.8 (15.6, 27.9)	5.1 ± 1.9 (1.3, 8.9)	0.55
LS-Di, %	7.1 (5.9, 8.2)	6.9 (5.4, 8.3)	−0.23 ± 0.75 (−1.7, 1.2)	<0.1
PTS-Di, %	13.6 (11.8, 15.3)	16.0 (10.6, 21.5)	2.5 ± 2.1 (−1.6, 6.6)	0.22
RS-Di, %	12.9 (10.9, 14.9)	13.3 (10.3, 16.2)	0.34 ± 0.83 (−1.3, 1.9)	0.69

*Data are means (95% confidence intervals). Abbreviations as in Table 1*.

### Inter-associations Between Volume- and Strain-Derived SDI Indices

**Figure 2:**
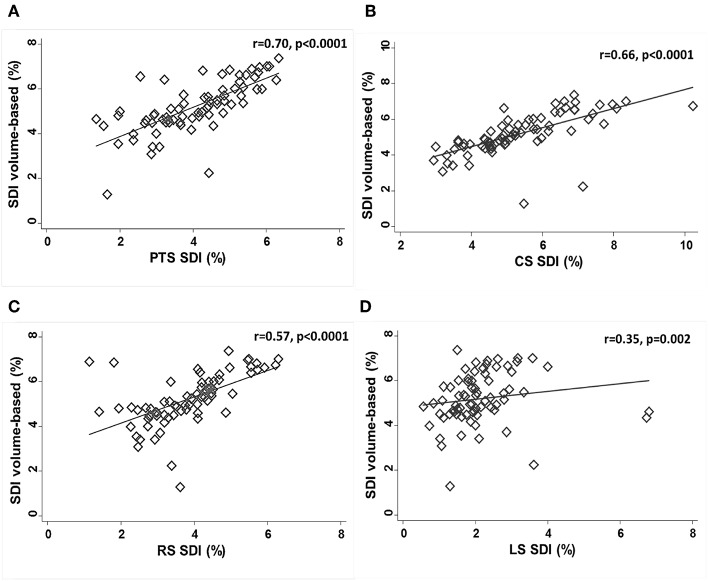
Correlations between SDI-volume and PTS-SDI **(A)**, CS-SDI **(B)**, RS-SDI **(C)**, and LS-SDI **(D)**. CS, circumferential strain; LS, longitudinal strain; PTS, principle tangential strain; RS, radial strain; SDI, systolic dyssynchrony index.

PTS-SDI and CS-SDI correlated well with SDI-volume (*r* = 0.70, *p* < 0.0001 for PTS-SDI; and *r* = 0.66, *p* < 0.0001 for CS-SDI) (Figure 2). RS-SDI correlated moderately with SDI-volume (*r* = 0.57, *p* < 0.0001), whereas LS-SDI correlated weakly (*r* = 0.35, *p* = 0.0025) (Figure 2).

## Discussion

3D-STE has theoretical advantages over other ultrasound-based methods for quantification of LVMD as it allows comprehensive evaluation of active contraction of the myocardium along different myocardial vectors simultaneously, and may therefore provide a better representation of LVMD (15). However, to be useful 3D-STE evaluation of LVMD needs to achieve acceptable reproducibility in the context of a clinically-relevant range of image qualities. We assessed the effect of image quality on test-retest reproducibility of 3D-STE derived LV dyssynchrony indices and assessed the correlation between strain-derived indices and SDI-volume. We found that sub-optimal image quality did not introduce a systematic bias on 3D-STE derived strain-based LV dyssynchrony indices, although there was some evidence of underestimation of volume-based dyssynchrony indices with poorer image quality. However, under optimal conditions only volume, CS and PTS 3D-STE derived dyssynchrony indices achieved fair to good test-retest reliability. Under suboptimal conditions the reliability of all 3D-STE LV dyssynchrony indices was poor.

SDI-volume by 3DE has previously been reported to be a feasible and reliable measure to assess LVMD (16, 35). In a large meta-analysis, 3DE demonstrated 94% feasibility for the assessment of LVMD, and SDI-volume showed good intra- and inter-observer reproducibility based on re-reading scans [interobserver ICC = 0.92 (95% CI 0.88, 0.95) and intraobserver ICC = 0.95 (95% CI 0.93, 0.97)] (16). However, the authors highlighted the lack of estimates of test-retest reliability which is the relevant measure for follow-up assessments of LVMD. We show that when image quality is optimal volume-based LV dyssynchrony indices have acceptable test-retest reliability (albeit noticeably lower than re-reading reliability), but that the test-retest reliability of volume-based LV dyssynchrony indices is substantially lower for sub-optimal images.

Comprehensive evaluation of LVMD from different myocardial vectors including LS, RS, CS, and more recently AS (AS; principal tangential strain or 3D-strain) may provide a better representation of the active contraction of the myocardium than volume changes (15). Despite this, there is currently a limited number of studies which have assessed the reliability of strain-based LV dyssynchrony indices by 3D-STE (15, 19–22). Comprehensive assessment of LVMD of all myocardial directions by 3D-STE has been investigated by Thebault et al. although the authors only provided a re-read reliability assessment of AS derived dyssynchrony indices (21); the test-retest reliability of these indices has not been previously assessed. We show that LV dyssynchrony indices based on CS and PTS have fair to good test-retest reliability only when images are optimal, reliability is generally poor for sub-optimal images. Our data add to previous observations by Russo et al. who showed that poor image quality impaired the reliability of LV dyssynchrony indices assessed by 3DE even when re-reading the same images (18). We suggest that use of reliability estimates based on re-reading scans are likely to be over-optimistic and propose that test-retest estimates are a sounder basis for estimating sample sizes required to examine changes in dyssynchrony indices in follow-up studies.

Different components of myocardial mechanics reflect the contributions of different layers of the myocardium (36); these may be differentially affected by the extent and etiology of disease (37). It is possible therefore that LV dyssynchrony indices of different myocardial vectors may show differential associations with 3D-STE volume-based dyssynchrony indices. We found that SDI-volume correlated well with SDI derived from either PTS or CS and correlated moderately with RD-SDI, while SDI-volume correlated weakly with LS-SDI. This could be consistent with these measures reflecting different myocardial activation patterns but equally the poor correlation between LS-SDI and SDI-volume could simply reflect the poor reliability of LS-SDI. Further work is required to establish whether differences in 3D-STE LV dyssynchrony indices have any prognostic importance.

### Limitations

We acknowledge a number of limitations in this study. We only examined short-term test-retest reliability of 3D-STE derived LV dyssynchrony indices, and how they are influenced by sub-optimal image quality in healthy individuals. The use of healthy individuals makes generalization to specific cardiac pathologies difficult but at least some of our findings are likely to be relevant to studies of diseased populations since sub-optimal image quality is more likely in unhealthy individuals. Image quality is only one factor influencing the clinical utility of 3D-STE in assessing LVMD. While our approach produced impairments of image quality that were qualitatively similar to that seen in older or more obese patients, echocardiographic images may impaired by other factors such as emphysema or surgical scars which may be more difficult to simulate. 3D-STE is also constrained by limited temporal resolution and the effect of frame rate on reproducibility was not studied as this has been described by others (30), but we ensured that an adequate and consistent frame rate was maintained in all studies to avoid any bias from this source. The study was performed by a single observer which is a strength in that it avoids the influence of inter-observer variability but limits its applicability to typical clinical practice. Our study was performed on healthy individuals with good image quality (before image degradation) to maximize our ability to detect effects. Similar studies on individuals with cardiac disease, particularly dyssynchrony, would be valuable in future. We used software from a single vendor−3D-STE LV dyssynchrony indices have been reported to be uninfluenced by hardware or software (16), but other 3D-STE measures are vendor-dependent (35); therefore, our results should not be assumed to generalize to software from other vendors.

## Conclusion

Under optimal conditions in healthy individuals, the reliability of volume-derived and some 3D-STE strain-based LV dyssynchrony indices by test-retest was fair to good, but reliability was substantially compromised by poor image quality. Previous studies using re-reading of images as a measure of reliability have probably over-estimated the reliability of 3D-STE derived LV dyssynchrony indices.

## Data Availability Statement

All datasets relevant to this study are included in the manuscript/Supplementary Files.

## Ethics Statement

This study was carried out in accordance with the recommendations of World Medical Association (WMA). All subjects gave written informed consent in accordance with the Declaration of Helsinki. The protocol was approved by the UCL REC.

## Author Contributions

Each author has contributed extensively to the manuscript. LA and AH conceived and designed the study. LA performed the data collection and the statistical analysis and drafted the manuscript. CP and AH assisted in data interpretation, critically reviewed, and amended the manuscript.

### Conflict of Interest

The authors declare that the research was conducted in the absence of any commercial or financial relationships that could be construed as a potential conflict of interest.
