# Supplementary material for: Reproducibility of Left Ventricular Dyssynchrony Indices by Three-Dimensional Speckle-Tracking Echocardiography: The Impact of Sub-optimal Image Quality
Source: Front Cardiovasc Med. 2019 Oct 10;6:149. doi: 10.3389/fcvm.2019.00149 (PMC6795682; doi:10.3389/fcvm.2019.00149)
Supplement: Supplementary file 1 [file Data_Sheet_1.docx]

Supplementary Material

| **Table S1. Bland & Altman Analysis of 3D-STE derived LV dyssynchrony indices by the image quality (study 1)** | | | | |
| --- | --- | --- | --- | --- |
|  | **Good 1 vs. Good 2** | | **Sub-optimal 1 vs. Sub-optimal 2** | |
|  | **Mean_Diff_ ± SD** | **95% LOA** | **Mean_Diff_ ± SD** | **95% LOA** |
| **SDI _volume_, %** | 0.02±0.83 | -1.6, 1.6 | -0.18±1.6 | -3.3, 2.9 |
| **CS-SDI, %** | 0.15±1.01 | -1.8, 2.1 | 0.94±1.5 | -1.9, 3.8 |
| **LS-SDI, %** | 0.04±0.79 | -1.5, 1.6 | 0.003±1.4 | -2.8, 2.8 |
| **PTS-SDI, %** | -0.09±1.2 | -2.5, 2.3 | -0.15±1.6 | -3.3, 2.9 |
| **RS-SDI, %** | -0.33±1.3 | -2.9, 2.3 | 0.07±1.6 | -2.9, 3.1 |
|  | | | | |
| **Di _volumes_, %** | -0.16±2.2 | -4.4, 4.1 | -0.64±5.5 | -11.4, 10.1 |
| **CS-Di, %** | 0.13±3.3 | -6.4, 6.7 | 4.0± 6.7 | -9.1, 17.1 |
| **LS-Di, %** | 0.23±2.9 | -5.6, 6.1 | 0.90±4.6 | -8.1, 9.9 |
| **PTS-Di, %** | 0.48±3.4 | -6.2, 7.2 | -0.39±4.9 | -10.0, 9.2 |
| **RS-Di, %** | -0.94±3.9 | -8.6, 6.7 | 0.71±5.8 | -10.7, 12.2 |

CS, circumferential strain; Di, dispersion (difference between minimum and maximum time to peak of measure over 16-LV segments normalized to cardiac cycle duration); LS, longitudinal strain; PTS, principle tangential strain; RS, radial strain; SDI, systolic dyssynchrony index.


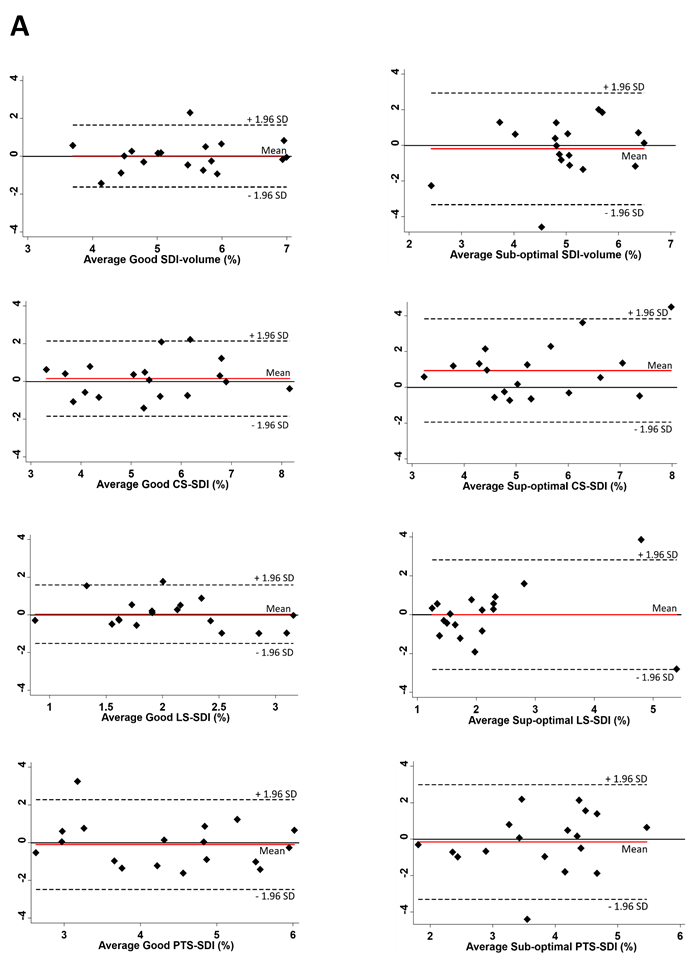


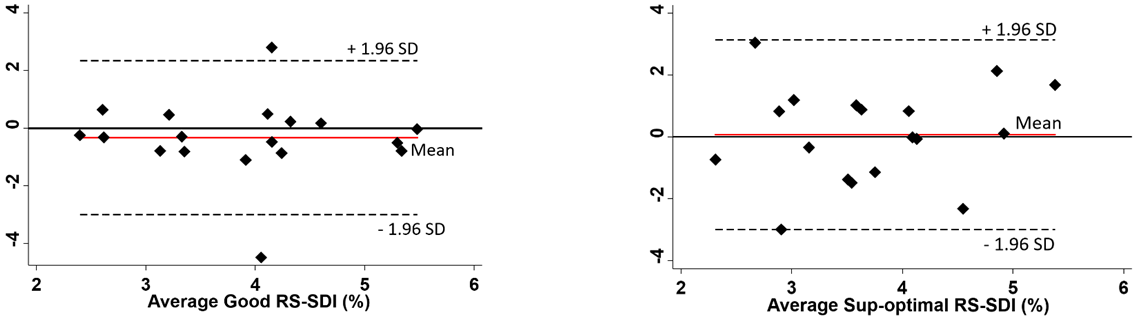


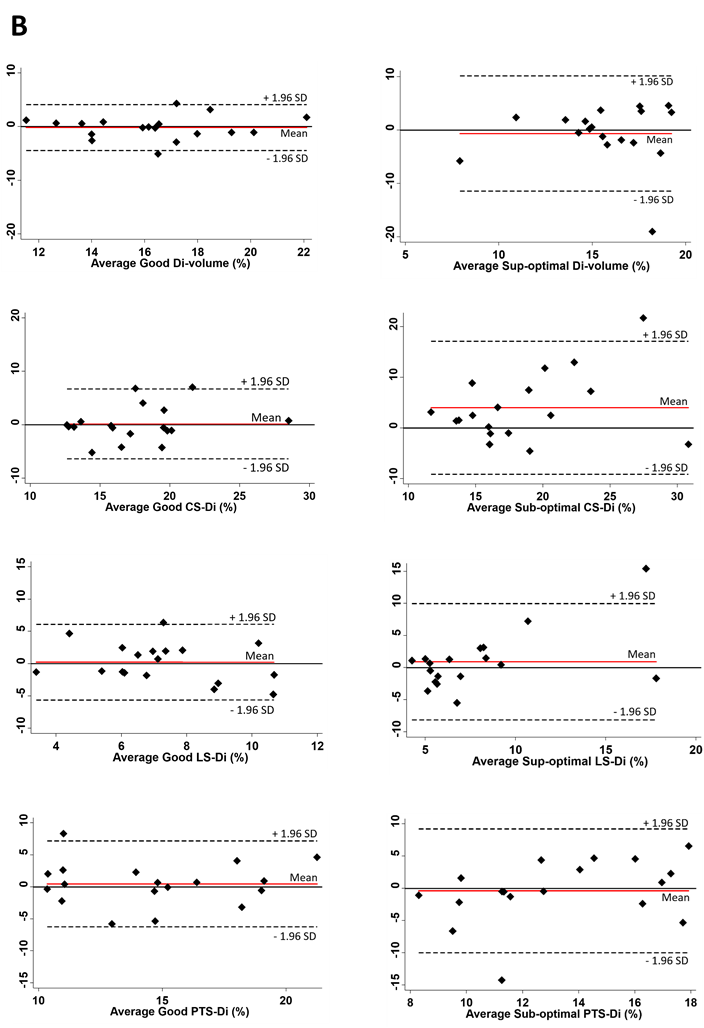


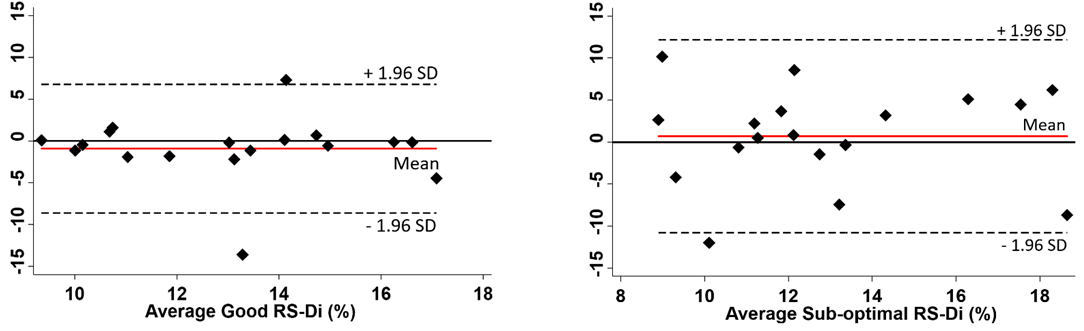


# Figure S1. Bland & Altman Graphs (A, B) of 3D speckle tracking echocardiography derived left ventricular dyssynchrony indices by the image quality (study 1).
